# Regulation of Tissue Responses: The TWEAK/Fn14 Pathway and Other TNF/TNFR Superfamily Members That Activate Non-Canonical NFκB Signaling

**DOI:** 10.3389/fimmu.2015.00092

**Published:** 2015-03-02

**Authors:** Linda C. Burkly

**Affiliations:** ^1^Department of Immunology, Biogen Idec, Inc., Cambridge, MA, USA

**Keywords:** non-canonical NFκB signaling, TWEAK, Fn14, TNF superfamily, TNFR superfamily

The immune system mediates tissue responses under both physiological and pathological conditions. In addition to leukocyte subsets, non-hematopoietic tissue cell types actively contribute to shaping tissue responses, including the inflammatory, fibrogenic, and regenerative components. TWEAK and its receptor Fn14, members of the TNF/TNFR superfamily, have emerged as a prominent molecular axis regulating tissue responses (1). Generally leukocyte-derived, TWEAK signals through Fn14, which is highly induced in injured and disease tissues on the surface of parenchymal, vascular, stromal, and progenitor cells, thereby orchestrating a host of tissue-shaping processes, including inflammation, angiogenesis, cell proliferation, and death, and regulation of progenitor cells. Of the downstream signaling pathways, particular attention has been given to the non-canonical NFκB pathway, given that TWEAK induces acute activation of canonical NFκB but prolonged non-canonical NFκB activation. Thus, non-canonical NFκB signaling may be a key mechanism underlying TWEAK/Fn14-induced tissue responses. The non-canonical NFκB pathway is known to play a role in immunity and disease pathologies and is typically activated by only a subset of TNFR superfamily members, including Fn14, TNFR2, BAFFR, CD40, LTβR, and RANK. Thus, there is also broad interest in the role of this subset of TNFR superfamily members and their downstream signaling potentials in the regulation of processes underlying tissue remodeling in health and disease. This Research Topic is addressed in a compilation of 19 expert reviews and 1 original research article.

The TWEAK/Fn14 pathway as an injury-inducible mediator of pleiotropic responses is introduced in a review of the work implicating sustained Fn14 signaling in disease pathogenesis and encompassing the current TWEAK/Fn14-targeting approaches for treatment of human disease (2). Broad relevance to neurological diseases is supported by a basic TWEAK/Fn14 role in regulating the structure and function of the neurovascular unit, thereby regulating blood–brain barrier (BBB) permeability (3). Furthermore, BBB damage appears to be an important component of neuropsychiatric systemic lupus erythematosus, and there is emerging evidence for a role for TWEAK/Fn14 in compromising the BBB in lupus (4). Also relevant to lupus is the pathogenic role of TWEAK/Fn14 in the renal manifestation of lupus nephritis. Indeed, evidence supporting TWEAK/Fn14-mediated pathological mechanisms in contexts of acute kidney injury and chronic kidney diseases is substantial and clinical targeting of TWEAK is ongoing in lupus nephritis (5). Also addressed in this Research Topic is the role of TWEAK/Fn14 in the pathological remodeling underlying other inflammatory diseases, namely cardiovascular diseases and obesity-associated Type-2 diabetes (6, 7), as well as in myocardial remodeling leading to heart failure (8), and a common theme also addressed in these articles is the potential use of soluble TWEAK as a biomarker for cardiovascular diseases. The expression of soluble TWEAK in biological fluids of patients with autoimmune/chronic inflammatory diseases and its potential as a biomarker of these diseases is also more broadly discussed (9). Given that TWEAK has emerged as a major cytokine regulating skeletal muscle biology, two articles are dedicated to its role in muscle wasting and mitochondrial dysfunction, and its complex regulation of myogenesis where distinct roles for canonical versus non-canonical NFκB signaling have been delineated (10, 11). Besides Fn14 upregulation in contexts of injury/disease, it is also highly expressed on tumor cells relative to normal tissue making it an attractive therapeutic target. Purcell et al. (12) review the growth inhibitory activity of an Fn14-specific antibody on an array of human tumor cells, differentially dependent on canonical and/or non-canonical NFκB, an approach that is currently being pursued as a novel cancer treatment.

In summary, there is substantial evidence implicating TWEAK/Fn14 in the regulation of physiological and pathophysiological tissue responses, though there is still an incomplete understanding of the role of TWEAK/Fn14-induced canonical versus non-canonical NFκB in these various contexts. Since soluble TWEAK and membrane TWEAK differ in their capacity to induce canonical NFκB, distinct biological responses may reflect spatial and temporal differences in sources of TWEAK (13). New studies continue to inform the understanding of TWEAK-induced signaling, including ubiquitination events that are key to orchestrating canonical and non-canonical NFκB activation (14).

The role of other TNF/TNFR superfamily members in shaping tissue responses in health and disease is also reviewed, concentrating on those that can activate non-canonical NFκB. In a review on TNFα signaling through TNFR2, Faustman and Davis (15) discuss the concept of leveraging TNFR2 agonism to reshape the T cell compartment in autoimmune disease, and to promote tissue regenerative processes. On the other hand, Gardam and Brink (16) review the importance of BAFF/BAFFR-mediated non-canonical NFκB signaling in peripheral B cell survival and maturation. Likewise, CD40L–CD40-mediated activation of non-canonical NFκB appears to be critical for B cell survival and possibly contributes to development of B cell malignancies (17). Both BAFFR and CD40-mediated non-canonical NFκB activation in B cells is restrained by the adaptor protein TRAF3, and the relationship between TRAF3 degradation and non-canonical NF-kB2 activation is delineated in an original research article (18). Beyond T and B cell activation, positioning cues are critical for proper immune system development and function. In this regard, LTβR plays a critical role in lymph node development and remodeling through its delivery of differentiation signals for reticular networks and vasculature (19). Tissue remodeling in the context of chronic liver diseases also features a prominent role for both LTβ and TWEAK in the crosstalk between liver progenitor cells and hepatic stellate cells, which can evolve into pathological fibrosis and hepatocellular carcinoma (20). Finally, Walsh and Choi (21) review the RANKL–RANK–OPG system, a preeminent player in the bone homeostasis, pathologies including mammary gland tumorigenesis, and in the interplay between bone and the immune system. This collection of expert reviews provides a current perspective of the role of this particular subset of TNF/TNFR family members that can activate non-canonical NFκB signaling in shaping tissue responses in contexts of development, homeostasis, and remodeling.

## Conflict of Interest Statement

Linda C. Burkly is a Biogen Idec employee and stockholder.
